# Comfort Evaluation of Wearable Functional Textiles

**DOI:** 10.3390/ma14216466

**Published:** 2021-10-28

**Authors:** Melkie Getnet Tadesse, Carmen Loghin, Ionuț Dulgheriu, Emil Loghin

**Affiliations:** 1 Ethiopian Institute of Textile and Fashion Technology, Bahir Dar University, Bahir Dar 608, Ethiopia; 2 Faculty of Textiles, Leather & Industrial Management, Gheorghe Asachi Technical University of Iasi, 700050 Iasi, Romania; cloghin@tex.tuiasi.ro (C.L.); ionut.dulgheriu@academic.tuiasi.ro (I.D.)

**Keywords:** wearable technologies, functional textiles, comfort evaluation, intelligent systems

## Abstract

Wearable E-textile systems should be comfortable so that highest efficiency of their functionality can be achieved. The development of electronic textiles (functional textiles) as a wearable technology for various applications has intensified the use of flexible wearable functional textiles instead of wearable electronics. However, the wearable functional textiles still bring comfort complications during wear. The purpose of this review paper is to sightsee and recap recent developments in the field of functional textile comfort evaluation systems. For textile-based materials which have close contact to the skin, clothing comfort is a fundamental necessity. In this paper, the effects of functional finishing on the comfort of the textile material were reviewed. A brief review of clothing comfort evaluations for textile fabrics based on subjective and objective techniques was conducted. The reasons behind the necessity for sensory evaluation for smart and functional clothing have been presented. The existing works of literature on comfort evaluation techniques applied to functional fabrics have been reviewed. Statistical and soft computing/artificial intelligence presentations from selected fabric comfort studies were also reviewed. Challenges of smart textiles and its future highlighted. Some experimental results were presented to support the review. From the aforementioned reviews, it is noted that the electronics clothing comfort evaluation of smart/functional fabrics needs more focus.

## 1. Introduction

Comfort is the most significant feature of materials that have close contact with human skin. Rossi [1] defined clothing comfort as a feeling or condition of pleasing ease, well-being, and contentment. He classified comfort dimensions as thermophysiological, psychological, and sensorial. Thermophysiological comfort is concerned with the heat balance of the body during various levels of activity, while psychological comfort is all about being at peace with oneself. Sensorial comfort is a fabric handle related to tactile, moisture, pressure, and thermal sensations [2]. Furthermore, some attempts have been made to give definitions in relation to clothing comfort. Here are some of them:

- A term related to the roles, values, and societal standing is the so-called physiological comfort [3];
- A state of harmony between the wearer and the surrounding environment [4]; and
- Balanced thermal regulation of the body—thermal comfort or a combination of physiological, psychological, and mental wellbeing of the human being [5].

All the said definitions are equally important in the aspects of clothing comfort. Comfort is a very fundamental and decisive factor for when people buy clothes. Knowingly or unknowingly, people check for physiological or psychological clothing comfort. Therefore, a fundamental understanding of clothing comfort, more specifically wearable functional textiles, is very important for quality of life.

**Figure 1:**
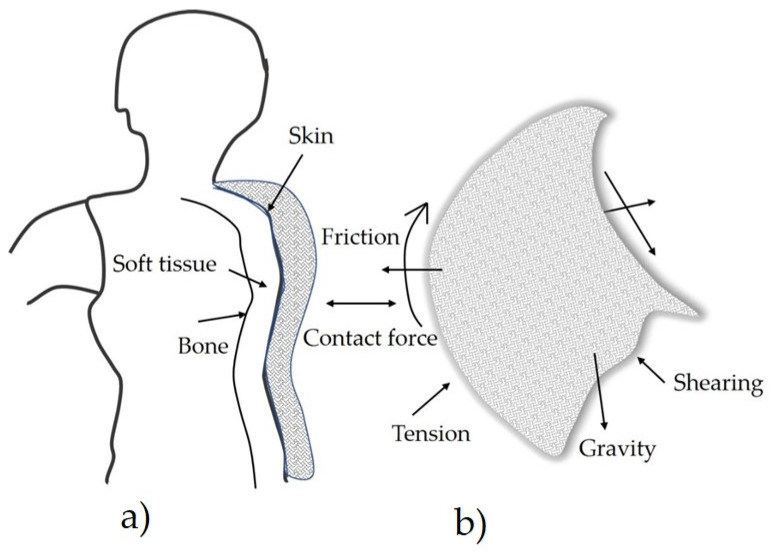
Illustrates where a rigid body (**a**) (assumed) is in continuous contact with a dynamically moving cloth (**b**). At first contact, the garment touches the skin; then, when the movement and friction tighten, it touches the soft tissues, and finally has the probability of disturbing the bone. The directions of the arrow on the cloth indicate the reaction of the cloth with the human skin. For example, the direction of gravity shows where the fabric has external forces beyond friction and contact with the skin.

Assessing the clothing comfort of clothing material is critical as there are many dynamic contacts between the clothing and the human body, such as tension force and bending of clothing occurs as the garment is worn on the human body where there are body parts brought the fabric to be bent or tensioned, as well as friction, compression, and some gravitational force against the human body where it stands. Figure 1 details various factors that contribute to discomfort of clothing. 

In a neutral environment, there is always a set friction between the garment and skin [6], gravity is exerted [7], there is shearing force due to the irregular body shapes [8], there is tensile force due to tensions, and elasticity and pressure have been exercised on the garment [9]. Compressional force is also not neglected [10]. All these clothing comfort issues are always naturally present between the worn garment and the human skin. Therefore, assessing the comfort of clothing materials whether conventional or e-textile is a required practice.

Clothing is one of the very general basics which the wearer requires to cover the body and protect it from very extreme surrounding conditions such as weather conditions. However, due to the various demands of the human being, clothing nowadays serves as protection, gives extra application, and is used for health monitoring, support in sport activities, and as a means of communications channels. Simply speaking, today, their names are preceded by a prefix called “smart” and thus are so called “smart textiles” or wearable textiles or functional textiles. The term smart textiles make things easier and simpler.

With the rapid developments in wearable technology and ever-increasing demands for electronic textiles (functional textiles), wearable electronics are becoming inadequate for use due to their heavy weight and non-flexible nature. They do have ridges and can press against the human body, bringing discomfort. Commonly, conventional clothing is expected to have a good ergonomic fit, be comfortable to wear, and be lightweight and flexible. Nowadays, due to the heavy weight of wearable electronics, the non-functionality of conventional clothing, and societal demands, smart textiles are now being widely developed [11,12,13]. Smart textiles or functional textiles are demarcated as textile constituents that are capable of changing their characteristic behavior with response to the inspiration of peripheral features or technical stimuli from the surrounding environment, with mechanical, thermal, electrical, chemical, or other external sources impacting the change [14,15]. The consumer-wearable electronics of today are past their commencement but are still very much in their early stages. One of the reasons could be comfort issues in different aspects. However, research on the comfort evaluation of wearable electronics is not growing fast when compared to research on wearable technology development and marketing. Most of the research on wearable electronics focuses on specific areas such as sensors, actuators, and electronic health record sharing systems (eHealth). The user most focuses on the benefits that are obtained from the functional fabric without worrying about clothing comfort.

Different from other works, this review paper gives insights into a synergy of electronics clothing comfort evaluation for different aspects of wearable electronics in general and wearable functional textiles in particular, beyond the other perspectives on their functions. Subjective, objective, and intelligent systems for comfort evaluation are addressed. The basic nature of functional textiles is not out of the range of conventional textiles; therefore, the review includes the work performed for basic textile definition.

## 2. Comfort Assessment of Wearable Electronics

**Figure 2:**
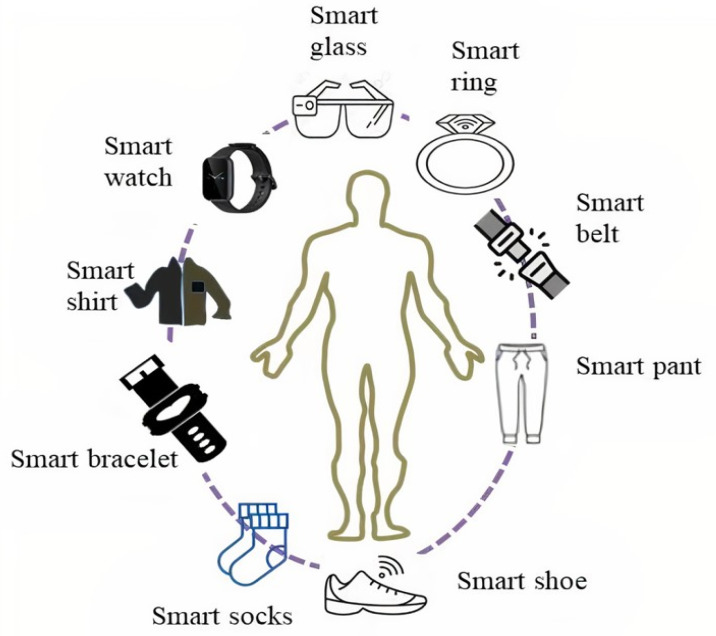
Schematic diagram of wearable electronics. All the wearable electronics illustrated have their own category of comfort dimensions such as physiological, tactile, thermal, and ergonomics aspects depending on physical structure and the place where they are fitted to the human body.

E-textiles are textile materials that have the following features: functionality, flexibility, wearability, being lightweight, and being able to interconnect sensors, actuators, and other electronic functions [16]. Possible trade-offs, such as ergonomics, fit, easy integration or interconnections, and low power consumption are also the main characteristics of e-textiles. Wearable electronics are illustrated as shown in Figure 2.

Intelligence or smartness is the third dimension of textile clothing [17]. The interactive dimension can be fulfilled using traditional production systems such as weaving [18], knitting [19], coating [20], and several other methods. Each construction technique has brought a change in the comfort values and hence its performance might be affected. Therefore, in order to obtain the full performance of smart textiles, wearing comfort has to be investigated.

In ergonomics, comfort can be assessed in various aspects such as thermal comfort [21], tactile comfort [22], and physiological comfort [23]. Comfort is a widespread problem in wearable electronics.

**Figure 3:**
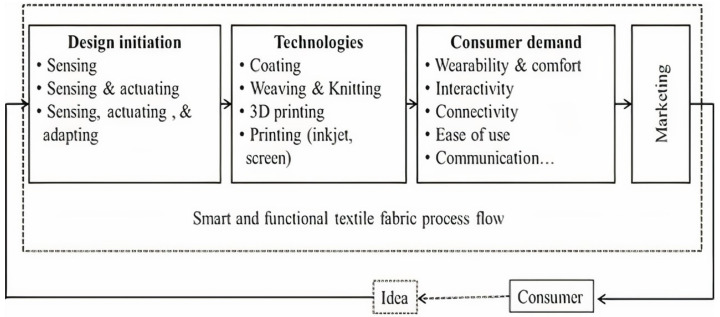
The smart/functional fabric development and marketing strategy flowchart. Here, only the fabric manufacturing process is presented. Garment assembly is an extra process not included in this schematic view. The basic dimensions in the smart clothing market value chain are listed.

E-textiles usually involve many processes from design to application. Figure 3 shows a simple view that helps to explain the parameters that should be fulfilled when designing smart and functional fabrics.

Functional textiles pass through complex processes such as weaving, coating, 3D (three-dimensional) printing, printing, and knitting. All these processes have their own effect on the comfort of functional textiles. It is well understood that finishing can alter the properties of fabric in many ways such as clothing comfort. Finishing is a complex process that adversely affects the surface characteristics of the fabrics. A number of changes occur during finishing treatments. The authors in Refs. [24,25] have discussed the effect of pigment printing on the softness of the product in an unfavorable way. On the contrary, Agrawal [26] has debated the effect of adding softeners on the hands of knitted fabrics in a favorable way. Low-stress mechanical properties have been measured by employing Kawabata evaluation systems (KES) and it was found that adding softener to the fabric benefits its mechanical properties, which has a direct relation with fabric comfort. However, it is not always true that various chemical reagents favor comfort. Other chemical reagents can adversely affect the fabric hand. For example, even though coating will change the conventional fabric into functional textiles, it has a negative effect on comfort [27,28,29]. Clothing comfort or fabric handle is one of the factors that may affect purchasing decisions. Therefore, care must be taken when thinking of functional textiles, as different treatment chemicals may affect their comfort values. Fabric handle is one of the important factors that affect clothing comfort. Fabric handle can be measured objectively [30] or subjectively [31]. One way or the other, measuring fabric handle leads to an understanding of clothing comfort. Total hand value (THV) or hand values (HV) of the clothing materials can be estimated either by stating good handle, average handle, or poor handle using qualitative means and by measuring the mechanical properties (using conventional machines) or measuring low-stress mechanical properties using Kawabata evaluation systems (KES) using quantitative means.

**Figure 4:**
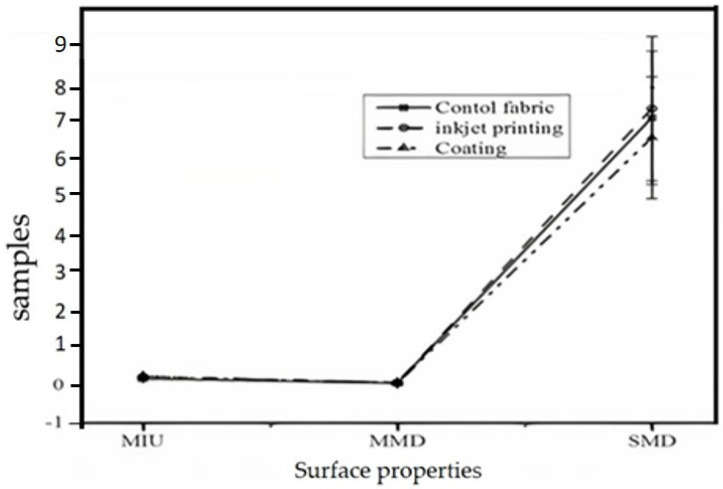
Surface-frictional properties of finished fabrics using different finishing techniques. The surface resistance of the conductive-coated sample is 7.98 kΩ/square while the surface resistance of the inkjet-printed samples is 0.168 kΩ/square. The coated sample was produced with a paint applicator at a gap height of 200 μm while the inkjet-printed samples were produced at 300 dpi and 50 layers. The samples were cured at 130 °C for 3 min. Average results were reported.

The surface properties of a conductive inkjet-printed and coated polyester fabric (substrate: plain woven fabric of type spun multifilament polyethylene terephthalate (PET) fibers with a weight of 158 g/m^2^; 30 ends cm^−1^; and 22 picks cm^−1^; scoured and heat-set by the supplier (Almedahl-Kinna AB, Boras, Sweden) sample coated and printed with conductive poly (3,4-ethylenedioxythiophene): poly (styrene sulfonate) (PEDOT: PSS)) were compared with the controlled fabric and the results are depicted in Figure 4. The surface properties of the functional fabrics were investigated using Kawabata evaluation system for fabrics (KES-FB-4) (Kato Tech Co., Ltd., Kyoto, Japan) where surface properties such as coefficient of friction (MIU—unitless), mean deviations of MIU (MMD—unitless), and geometrical roughness (SMD in µm) were determined and analyzed. The size of the sample was 20 cm × 20 cm which is in agreement with the Kawabata evaluation system (KES) standard [32]. The surface properties measurement was conducted to check the change in the roughness and smoothness properties of the functional fabrics after finishing.

The result indicated that functional finishing altered the geometrical roughness (SMD) of the functional fabric. However, when the two means were compared statistically, they were equal. Two sample comparison t-tests were performed in order to see the difference. The calculated t-stat and t-critical two-tail values for the coating vs. control were 3.08 and 2.78, respectively. The t-stat and the t-critical two-tail values for the conductive inkjet-printed specimen and the controlled sample were 1.90 and 2.78, respectively. In both cases, the null hypothesis (the two means are equal) was confirmed. Therefore, statistically, the effect of finishing on the handle of the textile product is not adverse for this treatment. However, clothing comfort is too sensitive and hence a little change in handle brings discomfort to the wearer.

Indeed, for wearable electronics, clothing comfort assessment has been carried out using various means such as subjective evaluation for running shoes [33] and personal protective equipment such as gloves, glasses, and elbow pads [34]. To enhance the performance of the user, the importance of clothing comfort is paramount. Radiation, evaporation, conduction, and convection are means of heat exchanges between the human body and the environment. Wearable electronics have an effect on thermophysiological, tactile, and ergonomic clothing comfort as each wearable electronics device hinders the heat exchange between the human body and surrounding areas. Therefore, clothing comfort evaluation of wearable computers has to be assessed as the discomfort has an effect on the efficiency of the user.

**Figure 5:**
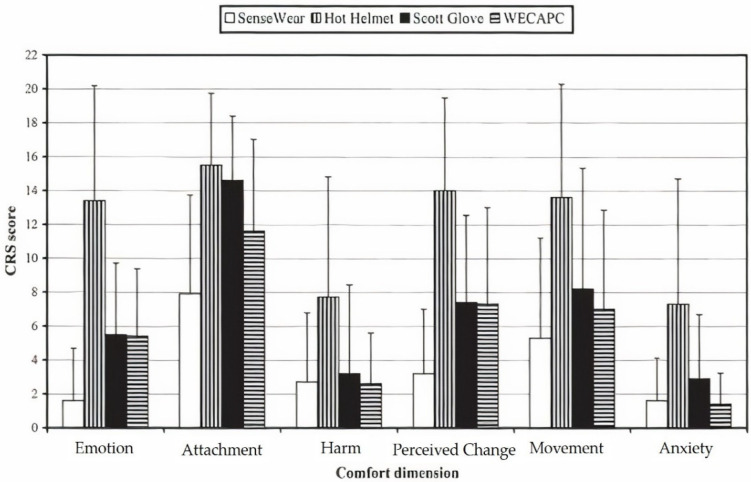
Comfort of wearable electronics across six dimensions, reprinted with permission from Ref. [35]. Copyright 2005 Sage publications. The result showed that multi-dimensional comfortable rating scales (CRSs) can be used to highlight differences in comfort between wearable electronics. WECAPC indicates web enhanced context aware personal computer.

Studies have been conducted to measure the clothing comfort of wearable computers, constructed on different dimensions such as anxiety, perceived change, injury, attachment, and feelings [35]. Subjective descriptor terms with different scales have been developed to predict the clothing comfort of different wearable electronics. Figure 5 illustrates results from various descriptors for different wearable electronics.

As observed in Figure 5, the attachment scores have the highest values, while anxiety and harm score the lowest values. The other multidimensional rating scales, perceived change, movement, and emotion, have scores between the two values. The values indicate that each CRS offered ratings for various aspects of wearable technologies and hence could affect wearable clothing comfort in different dimensions. Therefore, when thinking of wearable technologies, we must consider several variables, including multidimensional rating scales. The differences in the multidimensional rating scales provide some information regarding the comfort properties of various products.

A multidimensional assessment tool has been used to assess the clothing comfort of personal protective systems [36], while an ergonomic tool has been designed to assess medical equipment; the results showed that Steri-Shield provided the minimum discrepancy. Most of the wearable electronics are used to either treat patients or are used by doctors while examining the patients. A qualitative study [37] had to be carried out to assess the comfort of handheld computers and it was found that clothing comfort was the main building stone for their examination. Objective and subjective measurement of comfort for wearable computers was found to be a potential means as claimed by [38].

Clothing comfort is not only interpreted on tactile, thermal, and physiological dimensions but also with societal aspects [39]. More recently, wearable electronics have become increasingly dominant in the market. However, the societal perception of wearability in terms of comfort has not been touched. Social acceptance with respect to clothing comfort is as equally important as the functional aspects when qualitative studies are conducted.

In general, the comfort of wearable electronics can be assessed based on the conditions of appearance and relaxation (emotions), the tactile feel of the tool when touching human skin, the physical damage caused by the device, distress caused by the device, the ergonomics effect that affects the movement of the body, and the harm that the device will bring to the human body. However, all the aforementioned methods are qualitative and the result mainly depends on physical forms, age, gender, geographical location, and other environmental factors that are subject to biased results, which makes subjective evaluation difficult.

## 3. Comfort Assessment of Functional Textiles

**Figure 6:**
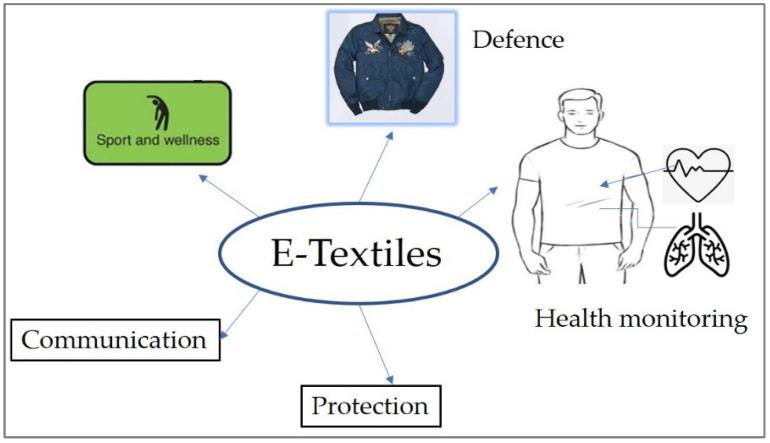
Various applications of smart textiles. Sport textiles are highly related to ergonomics comfort as movement is impacted, smart textiles for defense are susceptible to thermal comfort, smart textiles for health application are influenced by tactile and thermal comfort, and communication and protection smart textiles are based on their location on the human body.

Functional textiles are commercially accessible in various application forms such as health care [40,41], sport [42], military [43], communication [44], and protection [45], to mention a few. Figure 6 illustrates only a few applications of smart textiles that have direct contact with the skin.

Consumers that use wearable functional textiles always expect quality in terms of comfort when they are buying the materials. Therefore, wearable functional textiles should fulfill a wide range of comfort dimensions, including tactile, thermophysiological, societal, and other clothing comfort qualities to meet customer requirements. To this end, researchers utilize various clothing comfort quality evaluation methodologies such as subjective evaluation using expert systems, objective evaluation systems (using conventional physical properties measurement systems or using Kawabata evaluation or other instrumental means), and intelligent systems.

### 3.1. Subjective Evaluation Systems

**Figure 7:**
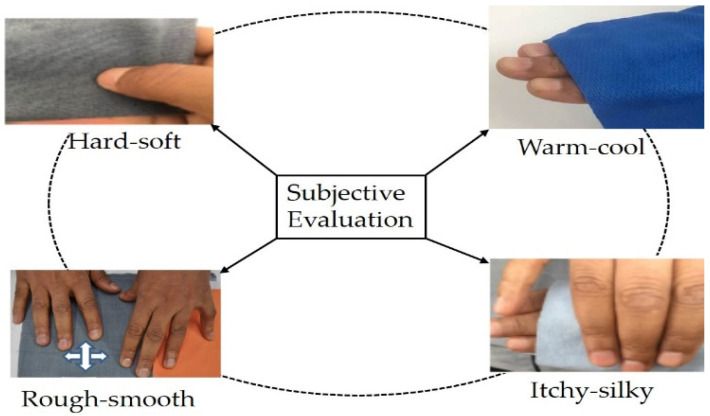
Fabric-skin-contact bipolar adjective pairs can be used to assess the comfort of functional textiles both blindly (where a box can be provided to hide the samples from the panelist’s vision) and visually (the panelist can see the object while assessing the samples. The panelist can say either the sample is hard or soft (in between—if scale is provided), warm or cold, itchy or silky, and rough or smooth.

Nowadays, the world wearable e-textile industry is progressively looking for new inventions to improve the fulfilment of the users in making persistent use of functional textiles for quality of life. However, clothing comfort affects the constant use of functional textiles. Therefore, comfort evaluation is a fundamental step. Subjective perception could be one alternative to assess the comfort of functional textiles. The subjective feeling of individuals can be analyzed by developing various sensorial bipolar terms to evaluate the comfort of functional textiles [46]. Certain fabric-skin-contact-related sensorial terms have been developed to assess the comfort of functional textiles by both blind and visual techniques and the result has showed that subjective evaluation could be one alternative for the evaluation of functional textiles comfort. Figure 7 shows how subjective evaluation can be used to assess the comfort of functional textiles by creating different bipolar descriptive words (sensorial terms that describe subjective clothing comfort in two extreme positions).

A possible way of assessing clothing hand during a user’s buying decision is either by observing the aesthetic design using visual sense or touching physically by hand. In both methods, clothing quality aspect for the buyer is fully sensorial. Technically, sensorial investigation of the textile-based product can be quantified by measuring or evaluating textile “hand or handle”. Fabric handle is a sensorial descriptor obtained when the fabric is touched by hand [47] or can be defined as a feeling observed when the fabric is rubbed, squeezed, or differently handled [48]. In both definitions, we can understand that fabric handle is related to some attributes when a fabric is handled in various ways. Attributes such as smoothness, roughness, scratchiness, slipperiness, softness, etc., can be described by fabric hand and assessed with different handling mechanisms. Fabric handle is a sensory phenomenon and hence fabrics are sensorial objects. For sensorial evaluation of the fabric comfort, the subjective assessment approaches are frequently used using human subjects [49,50,51]. The human subjects rate the handle of the fabric using sensory attributes that could help to define the handle of the fabric.

The comfort of ballistic vests (protective clothing) was studied using wear trials in 2009 [52]. The study affirmed that clothing fit and vest properties played a vital role in the comfort of the military clothing (functional protective clothing). However, to reach such conclusions, other information and data should be included, such as thermal comfort studies including other physical attributes.

Hand evaluation of textile-related goods using human perception has been performed using panels of experts [53]. Dijksterhuis [54] has classified panels of experts as field or street panels (consumers selected around shopping malls), consumer panels (consumers without any experience in which the evaluation is conducted in a controlled environment), and expert panels (those who receive required training). The number of panels may vary from one to several and should follow specified procedures, protocols, and conditions. They need careful control of the environment and should follow specific protocols.

The subjective rating scale varies depending on the final design analysis. For instance, Winakor et al. [55] performed a subjective evaluation using a 99-point semantic differential scale; the most common scale for hand evaluation is an eleven-point scale. The subjective assessment can be performed either by selecting 18 single or bipolar attributes depending on the researcher’s approach. The attributes should be carefully selected depending on the application of the textile product. For functional fabrics, physical attributes that can fully describe the handle of the functional fabric should be selected and the subjective evaluation should be carefully designed.

### 3.2. Objective Evaluation Systems

**Figure 8:**
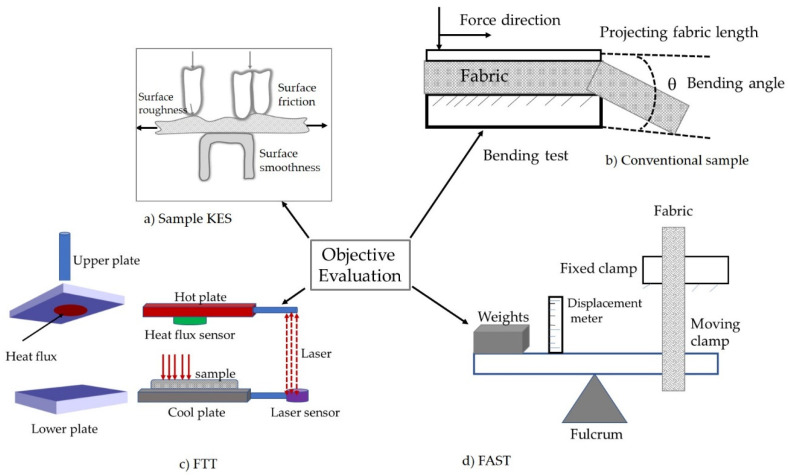
Methods of objective evaluation of fabric comfort. (**a**) Kawabata evaluation system (KES) where the effect of finishing on comfort-related properties has been assessed [57], (**b**) bending measurement of functionally finished fabric that has relation with comfort [58], (**c**) fabric touch tester (FTT) where comfort-related mechanical properties have been extracted [59], and (**d**) fabric assurance by simple testing (FAST) [60].

The comfort properties of functional fabrics have been predicted by measuring thermal conductivity, thermal resistance, thermal diffusion, and relative water vapor permeability [56]. Measuring the thermal-related properties of the functional textiles brought about the prediction of the thermal comfort of the garment, which is one method of comfort assessment by using objective means. Some of the methods are demonstrated in Figure 8.

Paek in 1975 [61] carried out an experiment on flame-retardant fabrics used for children’s sleepwear by measuring flexural rigidity, a coefficient of friction, and compactness, as well as the subjective evaluation using human panels. Roughness and openness showed better preferences than smoothness and compactness for the selected fabrics. However, the finding that smoothness is the most important factor for hand evaluation was disproven by Kawabata in 1980 [62]. Therefore, smoothness should be considered in sensory evaluation of smart clothing.

A study of the effect of printing on clothing comfort in 1997 [25] by Robinson et al. indicated that printing can change the comfort of clothing. The authors used a trained descriptive panel of experts to observe the effect of finishing (pattern and color) on the perception of clothing comfort using human subjects. One year later, in 1998, a study was carried out by Tzanov et al. [63] to examine the effect of finishing treatment on the fabric handle; it was confirmed that finishing could change the handle of the textile products in an unfavorable direction. The authors investigated the change in the fabric handle property of the finishing using KES methods. They found that the change in the bending property of the fabric after applying finishing treatment was high. This proved that measuring the low-stress mechanical properties has a relation with the handle of the fabric. On the other hand, the study indicated that Kawabata evaluation system (KES) can be applied for the hand evaluation of finished fabrics. KES systems have been used to investigate the comfort of functional fabrics such as conductive, thermochromic, electrochromic, and photochromic textiles [64]. The study investigated the effects of various finishing types by measuring tensile, surface friction, bending, shearing, and compressional properties which are associated with the comfort of functional fabrics. The authors claimed that the comfort of functional fabrics can be predicted by measuring the low-stress mechanical properties objectively.

A simple extraction method and mechanical properties measurement by American Society for Testing and Materials (ASTM) standards [65] have been applied to study the effect of functional finishing on the fabric handle. The study claimed that the extraction principle was an effective method for evaluating the total handle values of the fabrics resulting from different finishing types. In the extraction method, the load required to extract the fabric mainly depends on the bending and the surface friction properties only. The other mechanical properties such as tensile, shearing, and compression are not included in this method. Therefore, the extraction method is not the best method. That could be one of the reasons why most researchers have not applied this method for hand evaluation so far.

Later, in 2003, Cardello and his coworkers [66] studied the handle of a flame-retardant finished fabric that can be used for military clothing. They applied both the subjective (human expert) and the objective evaluation (KES) of the clothing material. A multiple regression model showed that good predictability of the handle of the military clothing was obtained from subjective and objective sensory evaluation methods. Even though the result looks promising, the multiple regression analysis may not fully give a better understanding of the relations between subjective and objective measurement results. This is because the relation between sensory data of fabrics is not linear but rather complex [67]. Researchers need more complex modelling techniques such as intelligent techniques to model the relation between sensory data.

In 2005, Yoo et al. [68] carried out a sensorial and thermophysiological comfort evaluation of protective clothing. The study was conducted to observe the effect of yarn type, weaving type, and functional finishing. They claimed that finishing had an influence on the comfort property of the protective clothing. Kawabata evaluation system (KES) was applied to measure the surface property of the protective clothing and hence evidenced that KES can be used to measure the comfort of the fabrics that have been functionally finished. The effects of coating and plasma treatment on the low-stress mechanical properties of fabric have been studied using KES measurement methods [29]. Similarly, the antimicrobial-coated cotton fabric has been studied using KES systems [28]. Both authors claimed that plasma treatment affected the handle of the treated fabric in a positive way while coating with different chemicals affected the handle in an unfavorable direction. However, both concluded that the functional finishing with coating applications can be assessed by measuring the low-stress mechanical properties using Kawabata’s evaluation system. KES is the well-known method to study the low-stress mechanical properties of fabrics which are most related to clothing comfort.

By the year 2008, Barker et al. [69] reviewed research that had been conducted on the assessment of functional clothing comfort. However, the work had a lot of limitations:

- The review was made based on a multilevel concept. That means the work was performed using only a wear trail base; however, there are a lot of methods for handle assessment such as subjective assessment by hand, objective assessment by instruments and so on;
- The review was only about thermophysiological comfort and sensorial comfort was not evaluated in detail and it was a very short paragraph for each section; and
- The authors concluded that combined human and instrumental data cannot provide sufficient information about the comfort of clothing. They claimed that material properties such as yarn property, fiber property, and finishing property may provide full information about the comfort of functional clothing. However, the study did not show details about studying such properties.

**Figure 9:**
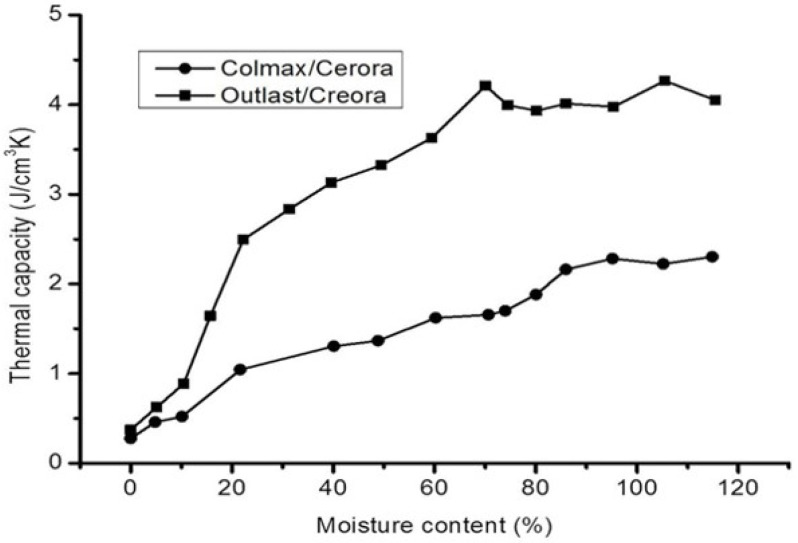
Effects of moisture content on thermal comfort of functional fabrics. Colmax^®^ is a thermo-regulating functional polyester yarn and Outlast^®^ is a thermos-regulating functional viscose yarn. Outlast has better moisture absorbency that that of Colmax, reprinted with per-mission from ref. [72].Copyright 2012 Sage publications. The better the moisture absorbency, the healthier its comfort.

Onal et al. in 2012 studied the thermal comfort of functional fabric using Alambeta and Permetest devices [70]. The study indicated that fabric design was the priority concern for thermal resistance and water vapor permeability while fiber composition played a vital role in the thermal absorption character (See Figure 9). However, thermal comfort is not only affected by design and construction, but finishing [71] and moisture [72] also have their own influence on thermal comfort. Finishing such as coating can reduce the moisture absorptivity of the fabric by filling the open pores between the yarns so that thermal comfort can be reduced. Therefore, it is not possible to define the factors that affect the handle of functional fabrics in a few parameters. It is a complex phenomenon that depends on multi-dimensions.

The surface properties of protective clothing have been studied using KES method [73]. In this article, the effects of physical properties, fabric construction, and moisture content have been explored. The authors claimed that the resiliency of the fabric could play a vital role in the sensory comfort of the protective functional clothing and it affects the surface property of the fabric. However, the study did not show the effect of moisture content and wetting on other mechanical properties such as tensile, shearing, bending, and compression.

In 2014 Shaid et al. [74] conducted research on the thermophysiological comfort of protective clothing which was made by incorporating superhydrophobic silica aerogel nanoparticles with wool-aramid composite fabric. They found that coating can reduce air permeability by 61.76% and increase thermal resistance by 68.64% with only 2% aerogel nanoparticle concentration. This study confirmed that finishing has a severe influence on the thermal comfort of functional fabrics. Therefore, when designing functional fabrics, comfort should be given priority so that the expected functions from the e-textiles can be obtained.

## 4. Intelligence Systems/Soft Computing Systems in Clothing Comfort Evaluation

Neuro computing, fuzzy logic, and other evolutionary algorithms such as artificial neural network (ANN) and adaptive neuro-fuzzy inference systems (ANFIS) which are used for the prediction, modeling, and optimization of textile properties are called soft computing systems or intelligent systems [75]. Human tactile sensations for the textile goods are very complex [76] and hence linear modelling cannot solve such relationships. The comfort of textile goods, which is closely related to the user’s perception and acceptance, has been currently studied and relationships between the physiological perception and the instrumental data have been estimated using various intelligence techniques. The most frequently used intelligence methods applied in the prediction of the tactile comfort of textile goods are fuzzy logic, artificial neural network (ANN), and adaptive neuro-fuzzy inference system (ANFIS). Settle et al. [75] discussed the importance of soft computing/intelligence techniques in the textile industry. The use of fuzzy logic has been used by many researchers recently after LA Zadeh’s pioneering work in 1965 [77]. Zadeh applied fuzzy logic for solving uncertainty or fuzziness or ambiguity of different parameters. The comfort of textile goods, particularly physiological perception, is full of uncertainty and fuzzy logic was the perfect fit for this concept. After Zadeh’s work, Zimmerman [78] and Mamdani [79] introduced the IF-THEN rules which support the ideas of Zadeh.

**Figure 10:**
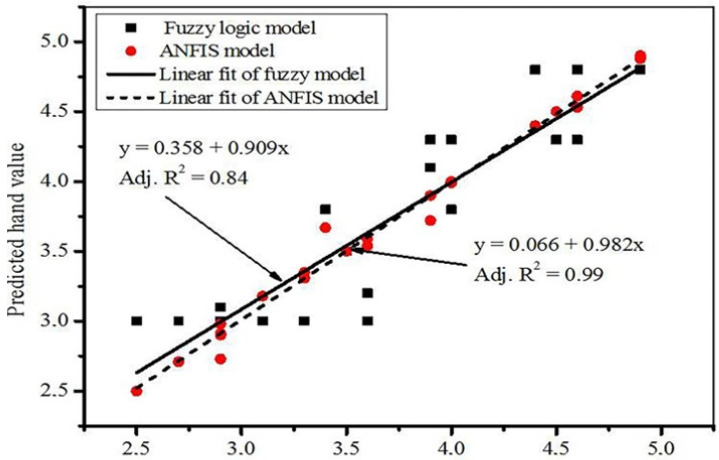
Comparison of fuzzy logic and ANFIS models on the prediction of the handle of knitted fabrics. The fuzzy logic model was taken without any modification from Jeguirim et al. [87] and the ANFIS model prediction result was performed using the same input parameters from Jeguirim and his co-worker’s work. The data were for the supple-rigid attribute only.

Thus, implementing fuzzy logic in the sensory evaluation of textile goods was started in 1991 by Raeel and co-workers [53]. The authors applied the fuzzy transformation matrix to predict the handle of lightweight dress fabrics and found good agreement between the subjective and the objective evaluation of the same fabric. The fuzzy transformation matrix enabled them to calculate the handle of the fabrics quantitatively, which further can be used as quality data which can be used for the communication between the user and the manufacturer. However, fuzzy logic has its own limitations as it does not have a specified technique that can be used as a mentor in the process of translating human perception into a rule-based fuzzy inference system (FIS) [80]. Other researchers [81,82,83,84,85] have applied fuzzy logic to predict and represent the comfort of textile goods. The conclusions from all authors confirmed that fuzzy logic is a better method for comfort prediction of textile goods when compared to linear statistical methods. However, the hybrid models, adaptive neuro-fuzzy inference system (ANFIS), can perform better than the fuzzy logic model. This is because ANFIS uses the FIS system to transform human knowledge into the rule-based fuzzy inference system [86]. To observe the difference between the efficiency of fuzzy logic and ANFIS, we used the work performed by Jeguirim et al. [87] on the use of the fuzzy logic model to predict the handle of knitted fabric from process parameters. We took the data from their work and we performed ANFIS using Matlab2017b^®^ and found the following results (see Figure 10).

A linear fit was employed to observe the difference between the two models (Figure 10). As observed, the linear model has a better fit for the ANFIS model. The values obtained by the fuzzy logic model were highly scattered. Hence, the ANFIS model performed better in predicting the handle of the textile goods.

The root mean square error (RMSE) and mean relative percent error (MRPE) were calculated for ANFIS and compared with the fuzzy logic model from Jeguirim and his co-worker’s work. These two parameters were used to evaluate the prediction performance of the models.

(1) $$RMSE=\sqrt{\frac{1}{N}}\overset{n}{\underset{i=1}{\sum }}{\left(p-y\right)}^{2}$$

(2) $$RMPE=\frac{1}{N}\left|\overset{n}{\underset{i=1}{\sum }}\left(\frac{\left(y-p\right)}{y}*100\right)\right|$$

where *N*; total number of observations, *y*; actual values, *p*; predicted values.

Based on this computation, the calculated RMSE, RMPE, and the standard deviation values for the ANFIS model were 0.083, 0.062, and 0.72, respectively, while the values according to the work of Jeguirim et al. were 0.29, 1.23, and 0.73, respectively. These results affirmed that the performance of the ANFIS model is higher than that of the fuzzy logic models. This is because fewer errors were observed in the case of the ANFIS model. Hence, this review paper recommends the ANFIS model over the fuzzy logic models. The adjusted R2 value is high. This confirms that the ANFIS model is the best model for predicting the handle of textile goods.

**Figure 11:**
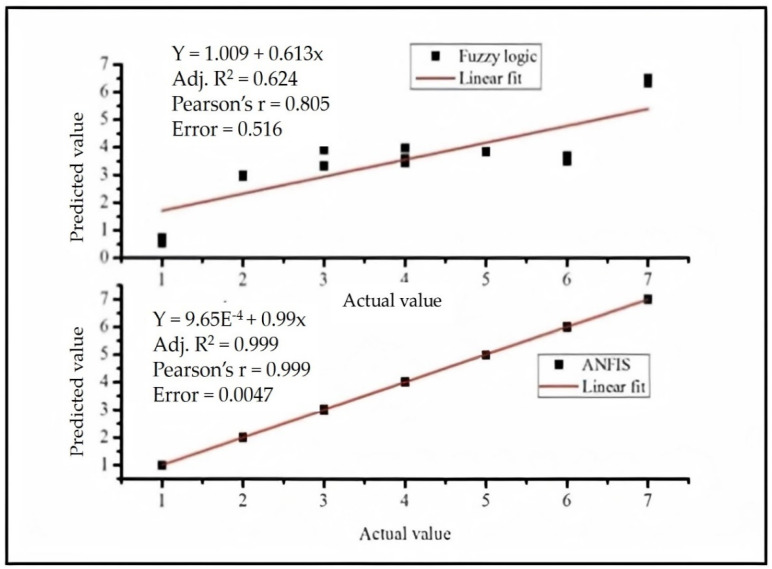
The performance difference between fuzzy and ANFIS models in the integration of human knowledge with the instrumental data. The input data from Zeng et al. [88] were taken as they are and we applied the ANFIS model to predict the handle of textile goods. The fuzzy logic model results are taken from Zeng et al. as they are for comparison purposes.

A similar review was made by Zeng et al. [88] (Figure 11). The researchers addressed the use of fuzzy logic to integrate human perception with the instrumental data on textile goods. They applied principal component analysis to support the extraction of fuzzy rules so that they could build the hand evaluation model. They found that fuzzy logic is an excellent model for such hand integration and estimation. We performed the ANFIS algorithm using the inputs from the paper and compared the results with fuzzy logic. We obtained the following result.

The results (Figure 11) clearly indicate that the ANFIS model is the best method for predicting the handle of the textile goods from instrumental and human perception data when compared to the fuzzy logic model. The calculated error based on the linear fit is much higher in the fuzzy logic model than in the ANFIS model. The Pearson correlation coefficient values indicated that the ANFIS model results are highly correlated to the actual values of the handle property of the textile goods. Therefore, even though fuzzy logic can be used to predict the handle of the textile goods, the best modelling is the ANFIS model. For this, a work performed somewhere else [67] discussed the application of fuzzy logic, neural network, data aggregation, classification, and clustering in predicting the handle of textile goods so that it can be used as a data source for quality inspectors, evaluators, and consumers for the clothing comfort quality check of their products. However, most recently, Xue et al. [89] have discussed advanced fuzzy logic techniques such as fuzzy genetic and fuzzy inclusion algorithms to predict human perception of the tactile comfort of textile goods. They claimed that the method they employed provides a good opportunity to analyze and interpret the vague qualities perceived by human knowledge. The work was also dedicated to showing how to assess fabrics using visuo-tactile scenarios which are equivalent to our daily experience, which the users used to assess the quality of the garment during buying decisions. They claimed that these intelligent techniques can solve the problems that exist in the classical computing techniques. The possible reason for this issue is that the soft computing techniques are used to develop problem-oriented descriptions and they are more flexible in nature.

Artificial neural network (ANN) is another intelligence method to estimate the comfort of textile goods. ANN has been used to predict the handle of textile goods from production and mechanical parameters [87,90,91]. It was claimed that the ANN model was slightly better than fuzzy logic models for calculating the RMSE and RMPE values of the predicted results. To this end, the optimization performed using principal component analysis (PCA) may decrease the performance of fuzzy logic. PCA is a technique for reducing the dimensionality of such datasets, increasing interpretability but at the same time minimizing information loss. It does so by creating new uncorrelated variables that successively maximize variance. Otherwise, the fuzzy logic and ANN models have comparative advantages for the sensory evaluation of goods [92].

An interesting and efficient method for the integration of human perception and instrumental data for the hand evaluation of textile goods is known as adaptive neuro-fuzzy inference system (ANFIS). ANFIS combines the advantages of ANN and fuzzy logic to transform human perception into interpretable data [93]. The ANFIS method can be used to construct the input-output mapping of human-based knowledge using the IF-THEN rules and then stipulate the data pairs. It uses the 3D and two-dimensional (2D) surface modelling system to forecast the input based on human knowledge to obtain the quantitative output data pairs. ANFIS has been used to study the subjective evaluation of some knitted fabrics by correlating sensory attributes and instrumental measurements [94] and subjective assessment of knit fabrics [86]. The researchers applied ANFIS to evaluate the subjective preferences for some attributes such as roughness and smoothness and they were able to relate the non-linear relationships between subjective preferences. They showed that ANFIS performs better than linear regression analysis methods. The merits of using ANFIS are its capability for predicting and integrating the non-linear relationships between physiological factors.

The objective data of functional fabrics from instrumental value [95] and the subjective assessment values from human experts [96] have been predicted using intelligent systems. Artificial neural network, fuzzy logic, and adaptive neuro-fuzzy inference system were implemented for clothing comfort prediction while the data were obtained both subjectively and objectively. The analysis indicated that the comfort of functional fabrics can be predicted using intelligence systems which make it easy to assess biased tasks. The actual predicted values show proximate reasoning.

## 5. Future Perspectives

Clothing comfort, more precisely, wearable textile comfort markedly influences not only our health, well-being, and work productivity, but also, we may lose the functional aspects. This review indicates that there is a noteworthy discrepancy in clothing comfort requirements due to diversified needs and various functional finishing aspects. Thus, studying the comfort of functional clothing needs to be profoundly investigated in advance. Smart/functional textile research has grown for the last two decades. However, the world market share is still at an infant stage. The possible reasons could be:

- There are few smart/functional fabrics on the market [97];
- There are no specific protocols or standards for the development and manufacturing of smart/functional clothing [98];
- The key factor for the quality of the product in terms of comfort is missing;
- The sensory evaluation of smart/functional clothing is currently investigated using methods developed for conventional fabric; and
- There are no standards for the comfort evaluation of smart/functional clothing.

On the other hand, there are a lot of research works being conducted on smart/functional fabrics such as WEALTHY, WearIT@work, MyHeart, MERMOTH, Avalon, Biotex, ProeTEX, Stella, OFSETH, Lidwine, and INTELTEX [99]. None of these projects deal with comfort issues but instead deal with the development and manufacturing aspect. Therefore, side by side, assessing the comfort of smart/functional fabrics should be given attention.

This review paper can be taken as a clue to researchers that quality evaluation and inspection of smart/functional fabric have been ignored. The future directions of this research field should be steered by combining development, manufacturing, quality control, inspection, and marketing strategy. Otherwise, in the future, smart/functional textiles may continue to have a very low market share in the world.

## 6. Conclusions

The hand evaluation of textile goods using sensory evaluation and the measurement of mechanical properties was introduced in 1930 while the effect of finishing techniques on the handle of textile goods was made familiar in 1975. The use of hand evaluation for the clothing comfort evaluation of conventional textiles as well as technically finished textile goods has been increasing and has become the topic for many researchers in the textiles field. Comfort evaluation of goods can be performed using subjective assessment by human experts or by measuring mechanical properties using various instruments. The integration of the subjective and objective data has been realized by mathematical and soft computing/intelligent techniques.

The application of soft computing/intelligent systems in hand evaluation of textile goods was introduced in 1991. Their use has been increasing for the last two decades. This is because the ability of soft computing to solve the non-linear relationships between the hand perceptions either through human judgment or using instrumental data has been effective.

It can be anticipated that in the future intelligent systems will continue to be used in the integration of human knowledge and instrumental data in the field of comfort science. On the way, some intelligent algorithms may be accepted as traditional comfort modelling tools for textile goods. Future research will probably shift its attention to the application of several soft computing/intelligent algorithms in the field of smart/functional textiles comfort modelling.

In general, this review work indicated that the most important dimension of smart/functional clothing, comfort, has been ignored and hence should be given attention in the future in order to increase smart/functional textile fabric acceptability.

## References

[1] Rossi R., Sr A. (2005). Interactions between protection and thermal comfort. Textiles for Protection.

[2] Gonca Ö., Kayseri N., Nilgün Ö., Gamze S., Jeon H.-Y. (2012). Sensorial Comfort of Textile Materials. Woven Fabrics.

[3] Kamalha E., Zeng Y., Mwasiagi J.I., Kyatuheire S. (2013). The comfort dimension; a review of perception in clothing. Sens. Stud..

[4] Slater K. (1986). The Assessment of Comfort. J. Text. Inst..

[5] Guowen S. (2011). Improving Comfort in Clothing.

[6] Gwosdow A.R., Stevens J.C., Berglund L.G., Foundation J.B.P., Stolwijk J.A.J. (1986). Skin Friction and Fabric Sensations in Neutral and Warm Environments. Text. Res. J..

[7] Lee J., Nam Y., Cui M.H., Choi K.M., Choi Y.L., Aykin N. (2007). Fit Evaluation of 3D Virtual Garment. Usability and Internationalization HCI and Culture UI-HCII 2007 Lecture Notes in Computer Science.

[8] Lindqvist R. (2016). On the relationship between the shear forces in human skin and the grain direction of woven fabric direction of woven fabric. Int. J. Fash. Des. Technol. Educ..

[9] Gong Y., Mei S. (2019). Stretch elasticity and garment pressure of shaping-underwear fabric Stretch elasticity and garment pressure of shaping-underwear fabric. Mater. Sci. Eng..

[10] Taylor P., Mak C.M., Yuen C.W.M., Ku S.K., Kan C.W. (2006). Objective evaluation of the Tencel fabric after fibrillation Objective evaluation of the Tencel fabric after fibrillation. J. Text. Inst..

[11] Shi Q., Sun J., Hou C., Li Y., Zhang Q., Wang H. (2019). Advanced Functional Fiber and Smart Textile. Adv. Fiber Mater..

[12] Koncar V., Cochrane C., Kelly F.M., Soulat D., Legrand X. (2018). Conductive polymers for smart textile applications. J. Ind. Text..

[13] Tao X., Tao X. (2001). Smart technology for textiles and clothing: Introduction and overview. Smart Fibres, Fabrics and Clothing.

[14] Koncar V., Koncar V. (2016). Introduction to smart textiles and their applications. Smart Textiles and Their Applications.

[15] Kirstein T. (2013). The future of smart-textiles development: New enabling technologies, commercialization and market trends. Multidisciplinary Know-How for Smart-Textiles Developers.

[16] Stoppa M., Chiolerio A. (2014). Wearable Electronics and Smart Textiles: A Critical Review. Sensors.

[17] Sungmee P., Sundaresan J. (2003). Smart Textiles: Wearable Electronic Systems. MRS Bull..

[18] Kunigunde C., Christoph Z., Thomas K., Niko M., Gerhard T. (2010). Woven Electronic Fibers with Sensing and Display Functions for Smart Textiles. Adv. Mater..

[19] Hao D., Xu B., Cai Z. (2018). Polypyrrole coated knitted fabric for robust wearable sensor and heater. J. Mater. Sci..

[20] Tadesse M., Loghin C., Chen Y., Wang L., Catalin D., Nierstrasz V. (2018). Effect of liquid immersion of PEDOT: PSS-coated polyester fabric on surface resistance and wettability. Smart Mater. Struct..

[21] Nilsson O.Ã. (2007). Thermal comfort evaluation with virtual manikin methods. Build Environ..

[22] Das A., Alagirusamy R., Song G. (2011). Improving tactile comfort in fabrics and clothing. Improving Comfort in Clothing.

[23] Bartels V., Shishoo R. (2005). Physiological comfort of sportswear. Textiles in Sport.

[24] Ozguney A.T., Taşkin C., Ünal P.G., Özerdem A. (2009). Handle Properties of the Woven Fabrics Made of Compact Yarns. Tekst Ve Konfeksiyon.

[25] Robinson K.J., Chambers E., Gatewood B.M. (1997). Influence of Pattern Design and Fabric Type on the Hand Characteristics of Pigment Prints. Text. Res. J..

[26] Agarwal G., Koehl L., Perwuelz A., Lee K.S. (2011). Interaction of textile parameters, wash-ageing and use of fabric softener during the laundry with mechanical properties of the Knitted fabrics and correlation with textile hand. I. Interaction of Textile parameters with Laundry Process. Fibers Polym..

[27] Zouhaier R., Mohamed H., Ayda B., Najeh M., Sadok R. (2013). Surface Roughness Evaluation of Treated Woven Fabric by Using a Textile Surface Tester. Res. J. Text. Appar..

[28] Kan C.-W., Lam Y.-L. (2013). Low Stress Mechanical Properties of Plasma-Treated Cotton Fabric Subjected to Zinc Oxide-Anti-Microbial Treatment. Materials.

[29] Lam Y.L., Kan C.W., Yuen C.W., Au C.H. (2011). Low stress mechanical properties of plasma-treated cotton fabric subjected to titanium dioxide coating. Text. Res. J..

[30] Pan N., Yen K., Zhao S., Yang S. (1977). Approach to the Objective Evaluation. Text. Res. J..

[31] Postle R., Mahar T. (1989). Measuring and Interpreting Low-Stress Fabric Mechanical and Surface Properties. Text. Res. J..

[32] Kawabata S., Behery H.M. (2005). The standardization and analysis of hand evaluation: The hand evaluation and standardization committee. Effect of Mechanical and Physical Properties on Fabric Hand.

[33] Bishop C., Buckley J.D., Esterman A.E., Arnold J.B. (2020). The running shoe comfort assessment tool (RUN-CAT): Development and evaluation of a new multi- item assessment tool for evaluating the comfort of running footwear. J. Sports Sci..

[34] Akbar-khanzadeh F., Bisesi M.S. (1995). Comfort of personal protective equipment. Appl. Ergon..

[35] James F., Chris B. (2005). A Tool to Assess the Comfort of Wearable Computers. J. Hum. Factors Ergon. Soc..

[36] Malik M., Handford E., Staniford E., Gambhir A.K., Kay P.R. (2006). Comfort assessment of personal protection systems during total joint arthroplasty using novel multidimensional evaluation tool. R. Coll. Surg. Engl..

[37] Mcalearney A.S., Schweikhart S.B., Medow M.A. (2005). Doctors experience with handheld computers in clinical practice: Qualitative study. BMJ.

[38] Pearson E.J.M. (2009). Comfort and its measurement. Disabil. Rehabil. Assist. Technol..

[39] Dunne L.E., Profita H., Zeagler C., Clawson J., Gilliland S., Do E.Y.-L., Budd J. The Social Comfort of Wearable Technology and Gestural Interaction. Proceedings of the 2014 36th Annual International Conference of the IEEE Engineering in Machines and Biology Society.

[40] Van Langenhove L. (2007). Smart Textiles for Medicine and Healthcare.

[41] Narbonneau F., D’angelo L.T., Witt J., Paquet B., Kinet D., Kreber K., Logier R. FBG-based smart textiles for continuous monitoring of respiratory movements for healthcare applications. Proceedings of the IEEE E-Health Networking Applications and Services.

[42] Ferraro V. Smart Textiles and Wearable Technologies for Sportswear: A Design Approach. Proceedings of the 2nd International Electronic Conference on Sensors and Applications.

[43] Bulgun E.Y. (2005). Smart Textiles for Soldier of the Future. Def. Sci. J..

[44] Gorgutsa S., Bachus K., Larochelle S. (2016). Washable hydrophobic smart textiles and multi-material fibers for wireless communication. Smart Mater. Struct..

[45] Langereis G.R., Bouwstra S., Chen W., Chapman R.A. (2013). Sensors, actuators and computing systems for smart textiles for protection. Smart Textiles for Protection.

[46] Tadesse M.G., Harpa R., Chen Y., Wang L., Nierstrasz V., Loghin C. (2018). Assessing the comfort of functional fabrics for smart clothing using subjective evaluation. J. Ind. Text..

[47] McIntyre I.D.P. (1995). Textile Terms and Definitions.

[48] Hoffman R.M., Beste L.F. (2015). Some Relations of Fiber to Fabric Hand. Text. Res. J..

[49] Vi A.Y.S. (2006). Sensory evaluation methods for tactile properties of fabrics. J. Sens. Stud..

[50] Kandzhikova G.D., Germanova-krasteva D.S. (2016). Subjective evaluation of terry fabrics handle. J. Text. Inst..

[51] Pense A.M., Guilabert C., Bueno M.A., Sahnoun M., Renner M. (2006). Sensory evaluation of the touch of a great number of fabrics. Food Qual. Prefer..

[52] Taylor P., Barker J., Black C. (2009). Ballistic vests for police officers: Using clothing comfort theory to analyze personal protective clothing. Int. J. Fash Des. Technol. Educ..

[53] Raheel M., Liu J. (2015). Empirical Model for Fabric Hand. Text. Res. J..

[54] Dijksterhuis G. (1995). Multivariate data analysis in sensory and consumer science: An overview of developments. Trends Food Sci. Technol..

[55] Winakor G., Kim J., Wolins L. (1980). Fabric Hand: Tactile Sensory Assessment. Text. Prog..

[56] Crina B., Blaga M., Luminita V., Mishra R. (2013). Comfort properties of functional weft knitted spacer fabrics. Tekst Ve Konfeksiyon.

[57] Namligöz E.S., Bahtiyari M.İ., Körlü A.E., Çoban S. (2008). Evaluation of Finishing Processes for Linen Fabrics Using the Kawabata Evaluation System. J. Test. Eval..

[58] Deng Y., Wang S., Wang S. (2016). Study on Antibacterial and Comfort Performances of Cotton Fabric Finished by Chitosan-silver for Intimate Apparel. Fibers Polym..

[59] Haji Musa A., Malengier B., Vasile S., Van Langenhove L. (2018). Practical Considerations of the FTT Device for Fabric Comfort Evaluation. J. Fash. Technol. Text. Eng..

[60] Tokmak O. (2010). Investigation of the Mechanics and Performance of Woven Fabrics Using Objective Evaluation Techniques. Part I: The Relationship Between FAST, KES-F and Cusick’s Drape-Meter Parameters. Fibres Text. East Eur..

[61] Paek S.L. (1975). Evaluation of the Hand of Certain Flame-Retardant Fabrics. Text. Res. J..

[62] Kawabatra S. (1980). The Standardization and Analysis of Hand Evaluation.

[63] Tzanov T.Z., Betcheva R., Hardalov I. (1998). Quality Control of Silicone Softener Application. Text. Res. J..

[64] Tadesse M.G., Nagy L., Nierstrasz V., Loghin C., Chen Y., Wang L. (2018). Low-Stress Mechanical Property Study of Various Functional Fabrics for Tactile Property Evaluation. Materials.

[65] Kim J.O., Slaten B.L. (1999). Objective Evaluation of Fabric Hand Part I: Relationships of Fabric Hand by the Extraction Method and Related Physical and Surface Properties and FAST techniques less suitable for industrial applications. Text. Res. J..

[66] Cardello A.V., Winterhalter C., Schutz H.G. (2003). Predicting the handle and comfort of military clothing fabrics from sensory and instrumental data: Development and application of new psychophysical methods. Text. Res. J..

[67] Zeng X., Ruan D., Koehl L. (2008). Intelligent sensory evaluation: Concepts, implementations, and applications. Math Comput Simul..

[68] Yoo S., Barker R.L. (2005). Textile Research Journal. Text. Res. J..

[69] Barker R.L. (2008). Multilevel Approach to Evaluating the Comfort of Functional Clothing. J. Fiber Bioeng. Inform..

[70] Onal L., Yildirim M. (2012). Comfort properties of functional three-dimensional knitted spacer fabrics for home-textile applications. Text. Res. J..

[71] Chung H.C.G. (2004). Thermal properties and physiological responses of vapor-permeable water-repellent fabrics treated with microcapsule-containing PCMs. Text. Res. J..

[72] Onofrei E., Rocha A., Catarino A. (2012). Investigating the effect of moisture on the thermal comfort properties of functional elastic fabrics. J. Ind. Text..

[73] Nawaz N., Troynikov O., Watson C. (2011). Evaluation of surface characteristics of fabrics suitable for skin layer of firefighters’ protective clothing. Phys. Procedia.

[74] Shaid A., Furgusson M., Wang L. (2014). Thermophysiological Comfort Analysis of Aerogel Nanoparticle Incorporated Fabric for Fire Fighter’s Protective Clothing. Chem. Mater. Eng..

[75] Sette S., Van Langenhove L. (2009). An Overview of Soft Computing in Textiles an Overview of Soft Computing in Textiles. J. Text. Inst..

[76] Sztandera L.M., Cardello A.V., Winterhalter C., Schutz H. (2013). Identification of the most significant comfort factors for textiles from processing mechanical, handfeel, fabric construction, and perceived tactile comfort data. Text. Res. J..

[77] Zadeh L.A. (1965). Information and control. Fuzzy Sets..

[78] Zimmerman H.-J. (1983). Using fuzzy sets in operational research. Eur. J. Oper. Res..

[79] Mamdani E. (1977). Application of fuzzy logic to approximate reasoning using linguistic synthesis. IEEE Trans. Comput..

[80] Suparta W., Alhasa K.M. (2016). Modeling of Tropospheric Delays Using ANFIS. SpringerBriefs in Meteorology.

[81] Wong A.S.W., Li Y., Yeung P.K.W. (2004). Predicting Clothing Sensory Comfort with Artificial Intelligence Hybrid Models. Text. Res. J..

[82] Luo X., Hou W., Li Y., Wang Z. (2007). A fuzzy neural network model for predicting clothing thermal comfort. Comput. Math. Appl..

[83] Zeng X., Koehl L. (2003). Representation of the Subjective Evaluation of the Fabric Hand Using Fuzzy Techniques. Int. J. Intell. Syst..

[84] Chen Y., Zeng X., Happiette M., Bruniaux P., Ng R., Yu W. (2009). Optimisation of garment design using fuzzy logic and sensory evaluation techniques. Eng. Appl. Artif. Intell..

[85] Lu J., Zhu Y., Zeng X., Koehl L., Ma J., Zhang G. (2009). A linguistic multi-criteria group decision support system for fabric hand evaluation. Fuzzy Optim. Decis. Mak..

[86] Ju J., Ryu H. (2006). A Study on Subjective Assessment of Knit Fabric by ANFIS. Fibers Polym..

[87] Jeguirim S.E., Babay A., Sahnoun M., Cheikhrouhou M., Schacher L., Adolphe D. (2011). The use of fuzzy logic and neural networks models for sensory properties prediction from process and structure parameters of knitted fabrics. J. Intell. Manuf..

[88] Zeng X., Koehl L., Sanoun M., Bueno M.A., Renner M. (2004). Integration of Human Knowledge and Measured Data for Optimization of Fabric. Int. J. Gen. Syst..

[89] Xue Z., Zeng X., Koehl L., Zeng S.T. (2018). To Multisensory Studies of Textile Products. Artificial Intelligence for Fashion Industry in the Big Data Era.

[90] Yu Y., Hui C., Choi T., Au R. (2010). Intelligent Fabric Hand Prediction System with Fuzzy Neural Network. IEEE Trans. Syst. Man Cybern. Part C Appl. Rev..

[91] Park S., Hwang Y., Kang B. (2000). Applying Fuzzy Logic and Neural Networks to Total Hand Evaluation of Knitted Fabrics. Text. Res. J..

[92] Ruan D., Zeng X. (2004). Intelligent Sensory Evaluation: Methodologies and Applications.

[93] Jang J.R. (1993). ANFIS: Adaptive-Network-Based Fuzzy Inference System. IEEE Trans. Syst. Man Cybern..

[94] Jeguirim S.E.G., Adolphe D.C., Sahnoun M., Douib A.B., Schacher L.M., Cheikhrouhou M. (2012). Intelligent Techniques for Modeling the Relationships between Sensory Attributes and Instrumental Measurements of Knitted Fabrics. J. Eng. Fiber Fabr..

[95] Tadesse M.G., Chen Y., Wang L., Nierstrasz V., Loghin C. (2019). Tactile Comfort Prediction of Functional Fabrics from Instrumental Data Using Intelligence Systems. Fibers Polym..

[96] Tadesse M.G., Loghin E., Pislaru M., Wang L., Chen Y., Nierstrasz V., Loghin C. (2019). Prediction of the tactile comfort of fabrics from functional finishing parameters using fuzzy logic and artificial neural network models. Text. Res. J..

[97] Cherenack K., Van Pieterson L. (2012). Smart textiles: Challenges and opportunities Smart textiles: Challenges and opportunities. J. Appl. Phys..

[98] Decaens J., Vermeersch O., Paricia Dolez V.I. (2018). Specific testing for smart textiles. Advanced Characterization and Testing of Textiles.

[99] Anne S., Lieva V.L., Philippe G., Denis D. (2010). A roadmap on smart textiles. Text. Prog..

